# Schmallenberg Virus: To Vaccinate, or Not to Vaccinate?

**DOI:** 10.3390/vaccines8020287

**Published:** 2020-06-08

**Authors:** Kerstin Wernike, Martin Beer

**Affiliations:** Institute of Diagnostic Virology, Friedrich-Loeffler-Institut, Südufer 10, 17493 Greifswald-Insel Riems, Germany; martin.beer@fli.de

**Keywords:** Schmallenberg virus, peribunyavirus, prevention, vaccination, cattle, sheep, goat

## Abstract

Schmallenberg virus (SBV), a teratogenic orthobunyavirus that infects predominantly ruminants, emerged in 2011 in Central Europe, spread rapidly throughout the continent, and subsequently established an endemic status with re-circulations to a larger extent every 2 to 3 years. Hence, it represents a constant threat to the continent’s ruminant population when no effective countermeasures are implemented. Here, we discuss potential preventive measures to protect from Schmallenberg disease. Previous experiences with other arboviruses like bluetongue virus have already demonstrated that vaccination of livestock against a vector-transmitted disease can play a major role in reducing or even stopping virus circulation. For SBV, specific inactivated whole-virus vaccines have been developed and marketing authorizations were granted for such preparations. In addition, candidate marker vaccines either as live attenuated, DNA-mediated, subunit or live-vectored preparations have been developed, but none of these DIVA-capable candidate vaccines are currently commercially available. At the moment, the licensed inactivated vaccines are used only to a very limited extent. The high seroprevalence rates induced in years of virus re-occurrence to a larger extent, the wave-like and sometimes hard to predict circulation pattern of SBV, and the expenditures of time and costs for the vaccinations presumably impact on the willingness to vaccinate. However, one should bear in mind that the consequence of seronegative young animals and regular renewed virus circulation might be again more cases of fetal malformation caused by an infection of naïve dams during one of their first gestations. Therefore, an appropriate and cost-effective strategy might be to vaccinate naïve female animals of all affected species before the reproductive age.

## 1. Introduction

In summer and autumn 2011, an unidentified disease characterized by fever, decreased milk production, and diarrhea was noticed in cattle in Germany and the Netherlands. The causative agent of the observed clinical signs, a novel orthobunyavirus of the Simbu serogroup (family *Peribunyaviridae*, order *Bunyavirales*), was eventually identified in blood samples of acutely diseased cows by next-generation sequencing-based metagenomics [1]. Based on the origin of the samples, a cattle farm located near the German city of Schmallenberg, the novel virus was named Schmallenberg virus (SBV) and is now the lead species for related viruses [2]. In keeping with other orthobunyaviruses, the tri-partite RNA genome of SBV encodes for a total of six proteins: the nucleocapsid (N) protein and a small non-structural protein (NSs) are encoded by the S-segment; the glycoproteins Gn and Gc, as well as a non-structural protein (NSm) by the M-segment; and the RNA-dependent RNA polymerase by the L-segment [3].

After its initial appearance near the German/Dutch border region, SBV spread very rapidly throughout Europe, causing large epizootics in the continent’s ruminant population [4]. Besides Germany and the Netherlands, SBV occurred in Belgium, France, Luxembourg, Italy, Spain, and Southern England within the first insect vector season, i.e., during the summer and autumn of 2011 [4,5]. Although the mechanism of overwintering is not known until now and despite a very high seroprevalence of approximately 70% to nearly 100% in ruminants in the initially most affected areas [6,7,8], SBV re-appeared in the following vector season [9]. In 2012, SBV spread across the British Isles to Scotland and Ireland [10,11,12,13,14], reached the Scandinavian countries [15,16,17] and Eastern Europe as far as Lithuania [18], crossed the Alps [19,20], and spread to the Mediterranean area [21,22]. Thus, the virus was detected Europe-wide only 1 year after its initial discovery [23,24]. Thereafter, it established an endemic status with a pattern of cyclic re-emergence to a larger extent every 2 to 3 years [25,26,27,28]. Thus, it has to be anticipated that SBV will re-appear regularly also in the future if no countermeasures are implemented and, therefore, this virus presents a constant threat for the ruminant population.

When considering potential preventive measures, one should take the transmission mode into account. Like all other members of the Simbu serogroup, SBV is transmitted by *Culicoides* biting midges [15,29,30,31,32,33,34]. Under Central European weather conditions, the peak season of the vectors responsible for virus transmission, i.e., the time of highest activity of the *Culicoides* biting midges, is during the summer and autumn months. 

## 2. Host Range and Clinical Manifestation

Since its initial detection in bovine samples, SBV has been found in several domestic ruminants, such as cattle, sheep, goats, and various captive and wild ruminants [26,35,36,37]. In addition, anti-SBV antibodies have been detected in further ruminant species in zoological parks, some other zoo animals, free-ranging wild boar, and a few dogs [38,39,40,41,42,43]. However, large-scale serological surveys performed in dogs in Belgium and wild carnivores in Germany did not provide any further evidence for SBV-infections of carnivores, as anti-SBV antibodies were not detected in any sample [41,44]. In addition, no SBV-specific antibodies were detected in free-ranging wild-type mice and shrews indicating that free-living shrews and rodents are most likely not susceptible to SBV-infection [41]. As some orthobunyaviruses can induce disease in humans [45,46,47], the possibility of SBV transmission to humans was one of the most important questions to answer at the beginning of the epizootic. Blood samples were collected from exposed human populations in Germany and the Netherlands and virologically and serologically investigated. SBV genome or specific antibodies against SBV were not detected [48,49]. Therefore, the public health risk was concluded to be absent or extremely low [49]. Hence, SBV affects predominantly ruminants.

In cattle, sheep and goats of all age groups, SBV induces either none or only mild unspecific clinical signs for a few days, associated with a short-lived viremia of 2 to 6 days [1,50,51,52]. However, when naïve pregnant animals are infected, the virus may cross the placental barrier and cause, dependent on the time of gestation when infected, abortion, premature birth, stillbirth, or fetal malformation. These malformations comprise a wide range of severity and include arthrogryposis, kyphosis, lordosis, torticollis, scoliosis, ankyloses, brachygnathia, mild to severe hypoplasia of the central nervous system, porencephaly, narrow spinal cords, or encephalomyelitis [53,54,55,56].

The susceptibility of the growing embryo or fetus to an infection and the associated clinical signs most likely depend on the maturity of the placentomes and fetal target organs and on the development of the fetal immune system. In small ruminants, the critical timeframe during which an infection might lead to malformation ranges from about 30 to 60 days after conception and in cattle from about 30 to 150 days of pregnancy [57,58]. 

## 3. Antibody Response

In ruminants of all age groups, anti-SBV antibodies are induced between 1 and 3 weeks after infection [50,51,52], and immunity acquired due to an earlier SBV-infection protects from re-infection [51]. In cases of prenatal infections, anti-SBV antibodies are present in the blood of the newborn before the intake of the colostrum of its mother [59,60], when the fetus has been infected after the development of its immune competence or when it has become able to develop specific antibodies during an ongoing infection. 

For other orthobunyaviruses, it was previously described that the viral N-protein elicits a strong humoral immune response [61], and the same holds true for SBV-infected animals [62,63]. Accordingly, anti-N antibodies are currently widely used for the serological detection of previous SBV-infections, especially because all commercially available ELISAs [64] are based on this protein. However, although anti-N antibodies are highly abundant, they do not have neutralizing activity [65]. In contrast, antibodies directed against the viral envelope glycoproteins, specifically Gc, neutralize SBV [65,66,67], which suggests a strong involvement of the glycoprotein in virus neutralization mechanisms, as has been previously observed also for other orthobunyaviruses [61,68,69]. In the case of SBV, sera from re-convalescent animals reacted only against the full-length Gc protein and its subdomains and not against Gn [66]. Therefore, the Gc protein is most likely the predominant, if not the only important, antigen for neutralizing activity. Hence, this protein has been selected among others for the design of subunit, live-vectored or DNA-mediated vaccine preparations. 

## 4. Impact of SBV

The negative economic impact of SBV originates mainly from stillbirth or fetal malformation [70,71,72], but also from the effect on adult animals [73,74] and trade restrictions that have been implemented in several non-affected countries [35]. Besides, the emotional well-being of animal owners is impaired due to the sight of a high number of dead and/or malformed newborns and because of the stress raised by the new disease [75,76,77].

The overall impact on adult animals is considered as limited, resulting mainly from dystocia, treatment costs in cases of birth complications, a slight reduction in fertility parameters, more frequent early embryonic deaths, and milk loss in dairy cows [72,73,74,78,79]. Nevertheless, due to a great fluctuation between herds, individual farms could have very high economic losses of several thousands of euros [73].

With regards to fetal infections, there seem to be differences between species, with significantly higher malformation rates in lambs than in calves [4,70]. In France, a malformation rate of about 3% was estimated in field-infected cattle during the 2012/2013 calving season, while in the same season on average 8% of lambs born in SBV-infected herds showed typical congenital malformation [70]. In Dutch dairy herds, an SBV malformation rate of even only 0.5% was calculated for calves born between February and September 2012 [78], and a German follow-up study revealed an equally low vertical transmission rate [59]. In contrast, in some sheep farms, more than 30 % of the pregnant ewes of a season developed abortion or malformation [80], a rate that was never reported from any cattle farm. Nevertheless, even such a low malformation rate as observed in the cattle population can play a role as an additional factor for economic damages in livestock farming, especially in naïve herds.

## 5. Preventive Measures 

Direct treatment options for SBV-infected animals are not available. However, due to the impact on animal welfare, animal production and the export of animals and their products [4,73,77,81], approaches for prophylaxis should be considered. For insect-transmitted pathogens like SBV, there are two main preventive measures: management strategies and vaccination. 

The use of insecticides or repellents could be taken into consideration to prevent potentially infected vectors from biting susceptible animals. However, a case-control study conducted in Germany provided no evidence for protective effects of those treatments [7]. Another option for preventing transplacental transmission of the virus to the developing fetus could be an intelligent breeding management system. The mating period could be adjusted in order to avoid that susceptible animals are in the critical phase of gestation during the season of the highest activity of the insect vectors responsible for virus transmission. A combination of such an adjusted breeding system with further protective measures like housing of susceptible animals and insecticide treatment might potentially result in a reduction of clinical cases [76,82]. Furthermore, when grazing is applied, the management could be adapted and youngstock kept outside during the major vector season, thereby exposing the youngstock to the vector, which might potentially lead to an SBV-infection before heifers or sheep conceive for the first time. As anti-SBV antibodies are detectable for several years after infection [83,84,85], the immunity acquired by a lamb or calf may prevent fetal infection during a later pregnancy. However, this concept requires the presence of infected insects every year and a very high transmission rate from the vector to the animal host to ensure that every young animal is bitten and infected. In reality, SBV has established a status of alternating low-level circulation and re-circulation to a larger extent every 2 to 3 years [25,26,27,28]. Therefore, a much more reliable way of prophylaxis than exposure to the bites of potentially infected insect vectors is needed and that could be vaccination, an approach that is discussed in more detail in the following sections. 

## 6. Successfully Tested Vaccine Preparations

A classical approach for vaccine development is the use of chemically inactivated whole-virus preparations, and such inactivated vaccine formulations indeed exist for further members of the Simbu serogroup. A Japanese multivalent vaccine against Akabane virus, Aino virus and the similarly teratogenic reovirus Chuzan virus has been developed and prevents reproductive disorders in immunized ruminants [86]. Unfortunately, this vaccine did not confer cross-protection against SBV [87], although serological cross-reactions between different members of the Simbu serogroup were described [88,89]. Nevertheless, based on this knowledge, SBV-specific, chemically inactivated vaccines have been developed in a short time frame and successfully tested in the major target species cattle and sheep [62,90]. Moreover, marketing authorizations have been granted for such inactivated vaccines (Table 1). Those vaccines were licensed for the British and French market in 2013, and in May 2015, such a vaccine received a marketing authorization for the entire European Union [91,92,93]. However, a major drawback of these safe and stable inactivated whole-virus preparations is the missing DIVA capability, i.e., the possibility to differentiate field-infected from vaccinated animals. Such marker vaccines provide several advantages over conventional whole-virus vaccines, first and foremost the possibility to demonstrate the freedom of disease by serological methods in the aftermath of an outbreak or to permit the safe movement of susceptible animals between affected and disease-free countries, thereby preventing economic losses due to the trade restrictions. The most promising DIVA-compatible antigen delivery systems include live attenuated vaccines, DNA-mediated, subunit or live-vectored vaccines [94]. All those preparations have been developed for SBV [63,67,95,96,97] and some of them were successfully tested in cattle, one of the major target species of SBV (Table 1). 

Taking successful approaches for further bunyaviruses such as Rift Valley fever virus (RFVF) as a basis [98,99], SBV mutant viruses lacking either NSs, NSm or both non-structural proteins in combination were generated by reverse genetics, comprehensively characterized in vitro and finally tested in a vaccination/challenge trial in cattle regarding their safety and protective efficacy [95]. While the virus lacking NSm induced viremia comparable to the wild-type virus, the vaccination with the NSs and the combined NSs/NSm deletion mutant viruses did not result in detectable virus replication. Moreover, the NSm- and NSs-deficient live-attenuated virus protected all immunized cattle from virulent virus challenge [95]. However, this candidate vaccine is currently not DIVA-capable, since discriminating diagnostic test systems are missing. In contrast, antibodies induced by immunization with newly developed Gc-based vaccines can be differentiated from those induced by infections with the wild-type virus by a combination of N-protein-based commercial ELISAs and neutralization tests. Thus, DNA-mediated subunit as well as live-vectored vaccines have been developed on the basis of Gc. While DNA-mediated vaccines have only been tested in small animal models until now [63,96], subunit or viral vector vaccines have proved their efficiency in the target species of SBV, specifically cattle [63,97]. 

For the design of the SBV-specific vector vaccines, two organisms previously described for vaccine development against other cattle diseases have been selected, namely equine herpesvirus type 1 (EHV-1) and the poxvirus modified Vaccinia virus Ankara (MVA) [97]. As an SBV-specific immunogene to be inserted into the viral vectors, the N-terminal domain of Gc that has recently been identified to be connected to virus neutralization [66,100], was selected [97], especially since it had already been successfully tested as a subunit candidate vaccine [63]. To summarize the tests of subunit or viral vector vaccines in brief, a multivalent antigen containing the covalently linked viral protein domains of SBV and the related Akabane virus expressed in mammalian cells and the MVA-based vector vaccine performed best by conferring complete protection in every immunized animal [63,97]. Hence, the N-terminal domain of the Gc protein may be suitable for use in SBV vaccines, however, its immunogenicity highly depends on the replication of the vector virus in the vaccinated animals and, in the context of subunit vaccines, on correct conformation and presentation [63,97,101]. Interestingly, when the domain was optimally presented, it provided a protective effect equivalent to that of the earlier inactivated vaccines or a live-attenuated vaccine [62,63,95,97]. Besides the Gc domain, further candidate targets for vaccine development were suggested [96], however, their efficiency testing in ruminants is still pending.

None of the aforementioned (DIVA-capable) candidate vaccines are currently commercially available. 

## 7. Recent Use of Vaccines and Potential Vaccination Strategies

To our knowledge, the licensed inactivated vaccines are currently only used to a very limited extent. As they received their marketing authorization only after the first infection wave in Central Europe, which by itself resulted in a very high seroprevalence [6,7,8,102], and antibodies acquired due to an earlier SBV-infection protect from re-infection [51], it was not considered necessary to vaccinate the animals once the vaccines became available. In regions not affected in 2011, but first hit by SBV-infections in one of the following vector seasons, the willingness to vaccinate appeared greater, at least in the years immediately following the SBV emergence. While, for example, in 2013 about 13% of British sheep farmers vaccinated their animals against SBV, this rate declined in the following years to the point where almost no vaccinations were reported in 2015 and 2016 [76]. Presumably, the sometimes unpredictable and wave-like nature of SBV circulation had impacted on the willingness of farmers to vaccinate. Although higher lamb mortality, dystocia and associated ewe deaths as well as a higher impact on animal welfare and farmer emotional wellness were reported by animal owners from affected herds [76,77], the vaccine uptake was poor, eventually leading to the withdrawal of the vaccine from the British market [76].

As mentioned above, it was not considered necessary to vaccinate the animals in regions with a high proportion of seropositive animals. This high seroprevalence rate acquired due to natural infections of the ruminant population presumably led to the only sporadic virus detections in the year 2013, predominantly in the young stock. However, in the following years the herd immunity declined since the livestock population dynamics strongly influence the duration of herd immunity. Due to the high rate of replacement of the seropositive animals by seronegative young animals under routine animal production conditions, the overall rate of seropositive animals declined, leading to a decrease in herd immunity [85]. This decline in herd immunity most probably facilitated the renewed virus circulation, which has been observed for instance in Germany in the years 2014, 2016 and 2019 [26,27,28]. Thus, it is very likely that SBV will persist in Europe with seasonal variations and epizootic peaks when countermeasures such as vaccination campaigns are not implemented. The consequence of regularly renewed virus circulation may be again more cases of fetal malformation caused by an infection of naïve dams during gestation. Hence, this animal group, i.e., young females, could be one target of vaccination regimes against SBV. As outlined earlier, an intelligent management system or an adjustment of the mating period could be likewise taken into consideration to prevent fetal SBV-infections. However, such strategies may be difficult to implement where production and management systems are geared towards market demands or the seasonality of grass growth, and the use of protective housing is often impractical for extensive livestock production systems. Moreover, the experience and knowledge gained since 2011 demonstrated that such strategies most likely have only a limited effect, since a larger number of SBV-induced malformations was seen regularly following virus circulations and the related infections of naïve pregnant dams. In contrast, vaccination might have a strong impact. In addition to being used by individual livestock owners to protect their animals from infection, it could also be employed more strategically to break the transmission chain and reduce the overall spread of the virus [103]. 

European experiences during the outbreak of the reovirus bluetongue virus (BTV) in 2006/2007 have already demonstrated that vaccination of livestock against a vector-transmitted disease can play a major role in reducing the virus circulation or even in eradicating the disease from some regions [104,105]. BTV is also transmitted by *Culicoides* biting midges and predominantly infects ruminants [106]. Based on the similarities between SBV and BTV regarding the insect vector species responsible for virus transmission and the affected host animals, the knowledge gained during the BTV outbreaks most likely applies to SBV as well. In 2006, BTV serotype 8 was reported for the first time on the European continent causing a massive, economically devastating outbreak in the ruminant population [107]. Although various control measures including obligatory indoor housing, treatments with insecticides and trade restrictions were implemented at the national and EU level, the virus reappeared in 2007 affecting large parts of Western and Central Europe [108]. The massive outbreak was ultimately controlled by animal movement restrictions and intensive vaccination [107]. Regarding movement restrictions to control vector-borne diseases, however, it has to be considered that they can be logistically challenging and economically devastating [109], but do not prevent virus-infected midges from spreading over long distances, e.g., by wind movement [107,110]. 

Therefore, the most effective veterinary measure in response to the European BTV outbreak was vaccination. Overall, more than 100 million animals were vaccinated throughout Europe and, as a result, the incidence of BTV infections decreased rapidly [105], demonstrating the great success of vaccination campaigns in eradicating vector-transmitted viruses. Also for RVFV, a bunyavirus that is more closely related to SBV than BTV, vaccination can be an effective tool of disease control. Based on a transmission model, it was shown that vaccination can be effective in mitigating the impacts of disease outbreaks as a sole intervention measure [111]. However, the knowledge gained during the European BTV outbreak is more valuable than that related to RVFV to deduce the effects of countermeasures for SBV, since RVFV is transmitted by mosquitoes instead of *Culicoides* biting midges and the transmission pattern of arboviruses are highly dependent on vector ecology. 

In terms of vaccination strategies against SBV-infections, one should consider the extent of the measures. Mass vaccination resulting in a high coverage proved to be highly effective against arboviruses, but a high coverage will be difficult, if not impossible, to achieve in the case of SBV when vaccination is voluntary, especially when the costs are not refunded, but have to be paid by the animal owners. As an example, the probability of seroconversion dropped below 10% when vaccination against BTV became optional in France after 2010, while about 80% of the animals were seropositive in December 2009 when vaccination was compulsory [112]. However, as has been demonstrated for BTV, also a lower coverage can decrease infection prevalence [113]. In the case of multi-species pathogens likes SBV, it might be even beneficial to vaccinate only some of the target species or, when clinical disease occurs predominantly in a certain age group, a risk-based immunization of this group could be considered [103,114]. For vector-transmitted viruses even only vaccinating animals kept on pastures might be a cost-effective option [115]. 

To investigate an optimal vaccine deployment for SBV, a stochastic mathematical model was developed and used to simulate the effect of different scenarios for Scottish ruminants [103]. In Scotland, a region with only sporadic cases of SBV, vaccine impact was shown to be optimal when the high-risk area in the south, from where virus introductions were expected, was targeted [103]. Furthermore, when it was assumed that insect vectors feed preferentially on cattle, the relative impact of vaccination could be optimized when only cattle were immunized [103]. However, in practice the impact of SBV infections, specifically of fetal infections, is greater in sheep than in cattle [70] and, besides economic losses due to trade restrictions, the impact in infected herds originates mainly from abortion, stillbirth or the birth of severely malformed fetuses [70,71,72]. 

Therefore, the most appropriate strategy might be to vaccinate naïve female animals of all affected species before the reproductive age, especially in years of only low-level virus circulation and an associated low seroprevalence in the young stock. 

## 8. Conclusions

The unexpected emergence of SBV demonstrates the constant threat that yet unknown insect-transmitted viruses suddenly appear in previously unaffected regions, probably driven by a rapid change in global climate, trade and travel habits. Following its introduction into Europe, SBV established an endemic status with re-circulations to a larger extent every 2 to 3 years. Hence, it represents a constant threat to the ruminant population when no countermeasures, in particular vaccinations, are implemented. Previous experiences have already demonstrated that vaccination of livestock against a vector-transmitted disease can play a major role in reducing the virus circulation or even in eradicating the disease from some regions. However, the intermittent nature of SBV circulation and the expenditures of time and costs for the vaccinations, especially when the costs have to be paid by the animal owners, presumably impact on the willingness to vaccinate. Hence, a high coverage will be difficult to achieve. However, one should keep in mind that the consequence of regularly renewed virus circulation in combination with seronegative young stock may be again more cases of fetal malformation caused by an infection of naïve dams during one of their first gestations. Therefore, the most appropriate and cost-effective strategy might be to vaccinate naïve female animals of all affected species before the reproductive age. 

## Figures and Tables

**Table 1:** SBV-specific vaccines tested successfully in the target animal species of the virus.

Type of Vaccine	Description	Animal Species	Reference or Trade Name
inactivated	chemically inactivated whole-virus preparations	cattle, sheep	[62,90]
		cattle, sheep	Bovilis SBV (MSD Animal Health)
		cattle, sheep	Zulvac SBV (Zoetis)
		cattle, sheep	SBVvax (Merial)
modified-live	NSs or combined NSs/NSm deletion mutant viruses created by reverse genetics	cattle	[95]
subunit	N-terminal domain of Gc or linked ectodomains of Gn and Gc expressed in mammalian cells	cattle	[63]
live-vectored	N-terminal domain of Gc delivered by recombinant equine herpesvirus type 1 or modified Vaccinia virus Ankara	cattle	[97]
